# ColVI myopathies: where do we stand, where do we go?

**DOI:** 10.1186/2044-5040-1-30

**Published:** 2011-09-23

**Authors:** Valérie Allamand, Laura Briñas, Pascale Richard, Tanya Stojkovic, Susana Quijano-Roy, Gisèle Bonne

**Affiliations:** 1 Inserm, U974, Paris, France; 2 CNRS, UMR7215, Paris, France; 3 UPMC Univ Paris 06 UM76, IFR14, Paris, France; 4 Institut de Myologie, Paris, France; 5 AP-HP, Groupe Hospitalier Pitié-Salpêtrière, UF Cardiogénétique et Myogénétique, Service de Biochimie Métabolique, Paris, France; 6 Centre de Référence Neuromusculaire Paris-Est, Institut de Myologie, Groupe Hospitalier Pitié-Salpêtrière, Paris, France; 7 AP-HP, Service de Pédiatrie, Centre de Référence Maladies Neuromusculaires (GNMH) Hôpital Raymond Poincaré, Garches, France

## Abstract

Collagen VI myopathies, caused by mutations in the genes encoding collagen type VI (ColVI), represent a clinical continuum with Ullrich congenital muscular dystrophy (UCMD) and Bethlem myopathy (BM) at each end of the spectrum, and less well-defined intermediate phenotypes in between. ColVI myopathies also share common features with other disorders associated with prominent muscle contractures, making differential diagnosis difficult. This group of disorders, under-recognized for a long time, has aroused much interest over the past decade, with important advances made in understanding its molecular pathogenesis. Indeed, numerous mutations have now been reported in the *COL6A1, COL6A2 *and *COL6A3 *genes, a large proportion of which are *de novo *and exert dominant-negative effects. Genotype-phenotype correlations have also started to emerge, which reflect the various pathogenic mechanisms at play in these disorders: dominant *de novo *exon splicing that enables the synthesis and secretion of mutant tetramers and homozygous nonsense mutations that lead to premature termination of translation and complete loss of function are associated with early-onset, severe phenotypes. In this review, we present the current state of diagnosis and research in the field of ColVI myopathies. The past decade has provided significant advances, with the identification of altered cellular functions in animal models of ColVI myopathies and in patient samples. In particular, mitochondrial dysfunction and a defect in the autophagic clearance system of skeletal muscle have recently been reported, thereby opening potential therapeutic avenues.

## Review

### Collagen VI: an important component of connective tissues

**Figure 1:**
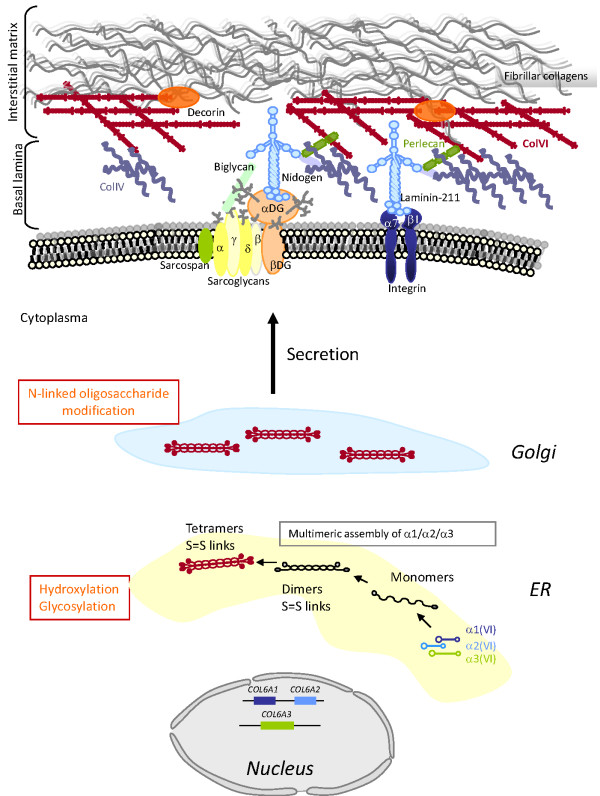
**Schematic representation of the collagen type VI (ColVI) intracellular assembly process, and interactions with skeletal muscle extracellular matrix (ECM) components**. Individual α(VI) chains fold through their triple helical domains to form monomers (1:1:1 ratio) in the endoplasmic reticulum (ER), which further align in an anti-parallel manner as dimers and tetramers that are stabilized by disulfide bonds between cysteine residues (S = S links). Post-translational modifications (indicated in orange) take place in the ER and Golgi, followed by secretion of tetramers that align non-covalently end to end, to form beaded microfibrils in the ECM. ColVI interacts with collagenous and non-collagenous components of the basal lamina and interstitial matrix surrounding muscle fibers.

Collagens are major constituents of the extracellular matrix (ECM), and are found in most connective tissues. They provide structural and mechanical stability to tissues, but they also play crucial roles in cell-ECM interactions through various receptors [1]. In particular, collagen type VI (ColVI), an important component of skeletal muscle ECM, is involved in maintaining tissue integrity by providing a structural link between different constituents of connective-tissue basement membranes (for example, collagen types I and IV, biglycan, and decorin) and cells [2-15] (Figure 1). In addition to its structural role, ColVI supports adhesion, spreading and migration of cells, and cell survival, as discussed later in this review.

ColVI is a heterotrimeric molecule composed of three individual α(VI) chains that display a similar structure, with a triple helical domain characterized by the repetition of the Gly-X-Y amino acid sequence, flanked by globular domains homologous to von Willebrand factor A domains [16,17]. In addition to the well-known α1(VI), α2(VI) and α3(VI) chains encoded in human by the *COL6A1, COL6A2 *(located head-to-tail on chromosome 21q22.3), and *COL6A3 *(on chromosome 2q37) genes [18], three novel chains, α4(VI), α5(VI) and α6(VI), have recently been identified [19,20]. These chains have high structural homology to the α3(VI) chain. In humans, the *COL6A4, COL6A5 *and *COL6A6 *genes are all located on chromosome 3q22.1, with the *COL6A4 *gene being split by a chromosome break and thus not coding for a protein [19-21]. The murine orthologs of these genes are organized in tandem on chromosome 9 (*Col6a4, Col6a5 *and *Col6a6*) and encode the α4(VI), α5(VI) and α6(VI) chains. The expression pattern of the three novel chains differs between mice and humans, and also between fetal and adult tissues [19,20]. Importantly, in the context of ColVI myopathies, the α6(VI) chain is the only one expressed at high levels in human skeletal muscle, at higher levels in fetal than adult tissue [19]. In skin, a detailed analysis of the expression of the human α5(VI) and α6(VI) chains revealed that both chains are expressed, albeit differently, and that they are variably altered in tissues from patients with mutations in the *COL6A1, COL6A2 *and *COL6A3 *genes [22]. Interestingly, the *COL6A5 *gene had previously been reported as associated with atopic dermatitis under the name *COL29A1 *[23], but this association has recently been questioned [24,25]. The knee osteoarthritis susceptibility locus *DVWA *was shown to correspond to the 5' part of the split *COL6A4 *gene [21].

Although largely ubiquitous, the expression of ColVI seems to be finely regulated in different cell types and tissues, as shown for the murine *Col6a1 *gene. The identification of a transcriptional enhancer located in the 5'-flanking sequence of the gene points to a collaborative crosstalk between myogenic and mesenchymal/endomysial cells, enabling transcription of ColV in muscle connective tissue [26-28].

The α1(VI), α2(VI) and α3(VI) chains assemble intracellularly as monomers (1:1:1 ratio), from their C-terminal ends, and subsequently form dimers (two anti-parallel, overlapping monomers) and tetramers (four monomers) that are stabilized by disulfide bonds between cysteine residues of the three chains [29-34]. ColVI chains are subjected to extensive post-translational modifications such as hydroxylation of lysine and proline residues [35], and glycosylation of hydroxylysines, which have been shown to be essential for the tetramerization and further secretion of ColVI [36,37]. Upon secretion, tetramers are further aligned end to end as microfibrils in the extracellular space, with a characteristic beaded appearance [33] (Figure 1). To date, somewhat contradictory results have been obtained regarding the possible assembly of the newly characterized α(VI) polypeptides with the α1(VI), α2(VI) chains. In transfection experiments, only α4(VI) appeared to have this ability [19], whereas in mouse muscle, all three were reported to do so [20]. Whether and how these additional chains may fit in the pathogenesis of ColVI myopathies remains unresolved to date, and needs to be addressed more comprehensively. To date, in our cohort of patients, no pathogenic mutations have been found by sequencing of the *COL6A5 *and *COL6A6 *genes in patients without mutations in the *COL6A1-3 *genes (V. Allamand, data not shown).

### Clinical phenotypes of collagen VI myopathies

**Figure 2:**
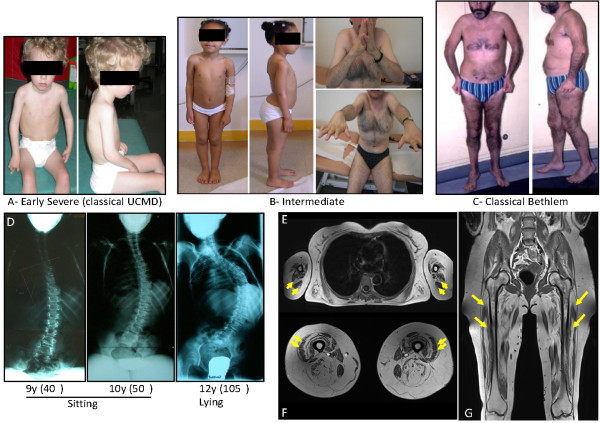
**Clinical spectrum, associated spine deformation and muscle MRI in collagen type VI (ColVI) myopathies**. **(A)**, Early severe phenotypes, corresponding to classic Ullrich congenital muscular dystrophy (UCMD), **(B) **intermediate forms seen in children or adults and **(C) **less severe, classic Bethlem myopathy (BM) forms constitute the overlapping clinical presentations of ColVI myopathies. **(D) **Radiography showing the evolution of spine deformation in a patient presenting with a classic early-onset UCMD phenotype. T1 transverse section of Bethlem myopathy upper limb girdle (E) and **(F) **thighs. Note the fatty infiltration, which appears as hyperintense area on T1-weighted images, located around the triceps brachialis muscles in (E) and along the fascia of vastus lateralis and vastus medialis muscles in (F) (yellow arrows). **(G) **The concentric fatty involvement of the thigh muscles is also seen on whole body MRI. (Images courtesy of Drs Susana Quijano-Roy and Tanya Stojkovic).

The etiological definition of ColVI myopathies as a specific condition has evolved over the years with the blurring of boundaries between two disorders, initially described separately but now recognized as the extreme ends of a continuous clinical spectrum [38,39] (Figure 2). The severe endpoint of this spectrum corresponds to Ullrich congenital muscular dystrophy (UCMD, OMIM 254090; http://www.ncbi.nlm.nih.gov/omim), described in 1930 as 'congenital atonic-sclerotic muscular dystrophy', emphasizing its early onset and the presence of proximal joint contractures associated with a striking distal hyperlaxity [40,41]. Orthopedic deformities (joint contractures, scoliosis) and respiratory impairment with diaphragmatic failure generally develop within the first decade of life, and may be life-threatening. Arrest of motor milestones with no acquisition of walking ability is seen in a subset of patients, but most children are able to walk, and show later progression of muscle weakness with loss of ambulation around 10 years of age, and a requirement for mechanical ventilation in late childhood or young adulthood [42,43].

At the other end of the spectrum is the milder form Bethlem myopathy (BM, OMIM 158810), described in 1976, which begins in the first or second decade, although a neonatal history may be recognized, characterized by early contractures of finger flexors, wrist, elbows and ankles [44,45]. Respiratory failure and distal hyperlaxity are usually absent or are milder than in UCMD, although the latter may not be so uncommon in very young children with BM. The course is usually slow, with most of the patients remaining ambulatory. However, progression of muscle weakness occurs often in the fifth decade, resulting in about 50% of patients requiring walking aids or a wheelchair [46]. Intermediate phenotypes have been described, and named 'mild UCMD' or 'severe BM', thereby reinforcing the notion of clinical overlap between Ullrich and Bethlem phenotypes [38,39].

Skin features such as follicular hyperkeratosis and hypertrophic scars or keloid formation are common [38,39,42,43,47-49]. Other common findings include normal cognitive abilities, normal or only slightly raised serum creatine kinase (CK) levels, and absence of cardiac phenotype. Two other conditions that fall within the spectrum of ColVI myopathies have been documented: autosomal dominant limb-girdle muscular dystrophy (LGMD) (in three families) and, more recently, autosomal recessive myosclerosis myopathy (OMIM 255600) (in one family) [50,51].

The prevalences of UCMD and BM in northern England has recently been reported as 0.13 and 0.77 per 100,000, respectively, amounting collectively to 0.9 per 100,000 [52]. UCMD seems to be the second most common type of congenital muscular dystrophy (CMD) in Europe (behind laminin α2 chain deficiency; OMIM 607855) and also in Japan (behind Fukuyama congenital muscular dystrophy; OMIM 253800, [53]) and Australia (behind α-dystroglycan glycosylation defects; [54]). In the cohort from northern England, BM emerges as the fourth most common myopathy behind myotonic dystrophy (OMIM 160900), facio-scapulo-humeral muscular dystrophy (OMIM 158900) and Duchenne/Becker muscular dystrophy (OMIM 310200 and 300376) [52].

### Differential diagnosis of ColVI-related myopathies

With the most prominent clinical presentation of ColVI myopathies being muscle weakness and contractures, associated with variable degrees of hyperlaxity, an important difficulty lies in defining boundaries and contiguities, with the possible differential diagnosis including congenital myopathies, Emery-Dreifuss muscular dystrophy (EDMD; OMIM 181350), LGMD, rigid spine muscular dystrophies, and other diseases of connective tissues such as Ehlers-Danlos syndrome [55-57]. Imaging techniques, such as computed tomography or magnetic resonance imaging (MRI) of muscle, are now recognized as very helpful in the diagnostic approach of muscle disease, because there are specific patterns of muscle involvement in each of these contractile myopathies as reported for EDMD with *LMNA *mutations [58], muscular dystrophies with rigidity of the spine [59], and ColVI myopathies [58,60,61]. From these studies, the typical pattern of muscle involvement in ColVI myopathies is now considered to be constituted by a diffuse, concentric hypodensity of the thigh muscles with relative sparing of the sartorius, gracilis and adductor longus muscles. The vasti muscles are the most affected muscles. In addition, a peculiar central area of abnormal signal is seen within the rectus femoris, initially referred to as a 'central shadow' [62].

In the context of the differential diagnosis, the absence of raised CK levels, the lack of a cardiac phenotype, and the presence of a specific MRI pattern are strongly suggestive of a ColVI myopathy.

### Molecular diagnosis and genetics

In light of the clinical variability and the overlapping presentation with other muscular disorders, a definite diagnosis can only be made after the identification of pathogenic mutations in one of the *COL6A *genes, which to date are restricted to *COL6A1*, *COL6A2 *and *COL6A3*. However, the large size (106 coding exons in total corresponding to 150 kb of genomic DNA) of these genes makes routine molecular diagnostics costly and time-consuming. The road to this 'holy grail' of diagnosis is thus often lengthy and full of pitfalls, and relies on a combination of clinical, biochemical and molecular findings.

**Figure 3:**
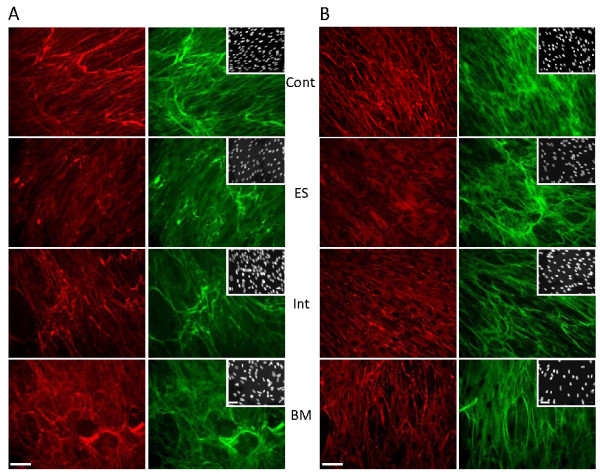
**Collagen type VI (ColVI) expression study in cultured skin fibroblasts**. **(A) **Representative images obtained using the protocol from [67] in which ColVI (red) is labeled with monoclonal antibody MAB1944 (Chemicon (now Millipore), Billerica, MA, USA) and perlecan (green) with monoclonal antibody MAB1948 (Chemicon). Note that ColVI expression appeared clearly altered in a patient with an early severe (ES) form and less so in patients with intermediate (Int) or Bethlem myopathy (BM) forms, compared with control fibroblasts (Cont). (B) Using the protocol of Hicks *et al*. [64], which detects ColVI (red) with polyclonal antibody Ab6588 (Abcam, Cambridge, UK) and fibronectin (green) with monoclonal antibody F15 (Sigma Chemical Co., St Louis, MO, USA), the sensitivity of the method is increased, and defective ColVI secretion could be detected in all patients' samples. Insets indicate nuclei, labeled using DAPI. Bars are 50 μm. (Images courtesy of Corine Gartioux and Valérie Allamand).

Historically, muscle biopsies were the routine and primary step undertaken for diagnostic purposes, and double immunostaining with a basement-membrane marker enabled recognition of ColVI deficiency in patients with UCMD [63], but not in patients with BM. The current diagnostic method of determining ColVI involvement is primarily based on immunocytochemistry of cultured skin fibroblasts, but this analysis is only available in a limited number of laboratories to date. A number of antibodies recognizing human ColVI are now commercially available and may be used for such techniques; in particular, the refined protocol proposed by Hicks *et al*.[64], using a polyclonal antibody raised against mature ColVI from human placenta, has better sensitivity, especially in fibroblast cultures from patients with BM (Figure 3). The absence or alteration of ColVI secretion in cultured fibroblasts, associated with clinical symptoms compatible with a diagnosis of ColVI myopathy, certainly warrants further genetic analysis.

**Figure 4:**
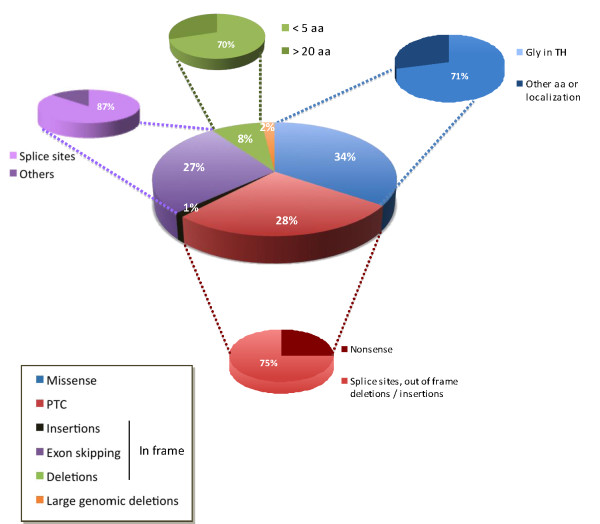
**Repartition of the various types of mutations identified in the *COL6A1, COL6A2 and COL6A3 *genes**. This schematic reflects, to the best of our ability, the distribution of 258 allelic mutations (98 on *COL6A1*, 113 on *COL6A2 *and 47 on *COL6A3*). Dominant *de novo *mutations represent 67% of the missense mutations, affecting glycine residues in the TH domains (Gly in TH), 57% of small deletions (< 5 amino acids) and 44% of splice-site mutations leading to in-frame exon skipping, whereas 97% of mutations leading to premature termination codons (PTCs) are familial (recessive or dominant).

Over the past decade, the development of genetic studies has demonstrated the heterogeneity and complexity of the molecular mechanisms at play in ColVI myopathies. An autosomal recessive pattern of inheritance was initially thought to be involved in UCMD, and linkage analysis led to the identification of mutations in the *COL6A2 *and *COL6A3 *genes [65-67]. However, numerous dominant *de novo *mutations have now been shown to be involved, accounting for more than 50% of the mutations causing UCMD [38,39,42,68]. Similarly, autosomal dominant mutations were first identified in the *COL6A1 *and *COL6A2 *genes in families with BM, suggesting that BM was mostly familial and inherited as an autosomal dominant disease [69], although rare *de novo *mutations and autosomal recessive mutations have now been reported [70-73]. To date, over 200 mutations have been identified in these genes, mostly distributed in the *COL6A1 *and *COL6A2 *genes. The most common types of mutations are point mutations, and mutations leading to premature termination codons (PTCs) and exon skipping (Figure 4). Among the former, missense changes affecting glycine residues in the triple helical domains of the corresponding proteins are the most common, and are often dominant *de novo*. Because these changes affect crucial amino acids within the collagenous domains, they hamper triple-helix formation [74-77]. Splice mutations resulting in in-frame exon skipping are generally dominant *de novo *mutations, and exons 16 of *COL6A3 *and 14 of *COL6A1 *seem to be preferentially affected, leading to UCMD or BM phenotypes, respectively [53,75,78-83]. Nonsense mutations and small deletions or insertions inducing PTCs within the coding frame are mostly inherited as recessive mutations, and lead to loss of function of the protein [42,53,68,75,76,79-92]. These mutations are responsible for most UCMD phenotypes. Nevertheless, it should be noted that genotype-phenotype correlations are very difficult to identify.

It has recently been shown that all types of mutations alter transcript levels, and that in the case of PTC-bearing transcripts, which are specifically degraded *via *the nonsense-mediated mRNA decay (NMD [93,94]) pathway, quantification of the three COL6A mRNAs is a helpful tool to pinpoint the mutated gene, thereby facilitating these cumbersome molecular analyses [42]. The NMD-induced degradation of PTC-bearing transcripts may also, at least in part, explain why the parents of patients with UCMD who themselves harbor recessive mutations are asymptomatic; their heterozygous status sustains the expression of 50% of the 'normal' protein, thereby leading to a 'functional loss of heterozygosity'. The study by Briñas and collaborators also provided some genotype-phenotype correlations in a cohort of patients with early-onset ColVI myopathy, showing that recessive mutations leading to PTC were associated with severe phenotypes [42]. Genetic studies are further complicated by a possibly variable penetrance as reported by Peat *et al*. [89].

Finally, the highly polymorphic nature of the *COL6A *genes makes it difficult to definitely assign pathogenicity to some variants, especially missense ones that do not affect glycine residues within the triple-helix domains of the proteins. In addition, these 'polymorphisms' may very well play a role in the extreme clinical variability of these conditions, particularly in patients carrying identical mutations but presenting with variable severity.

The types of mutations identified also reflect the methods used in laboratories performing these analyses (for example, sequencing of genomic DNA or of the coding sequences on cDNA), but the emergence of high-throughput methods (arrays) is likely to allow the identification of as yet unknown or under-recognized pathogenic mechanisms, such as large gene rearrangements, or promoter or deep intronic mutations, as recently illustrated in two reports [95,96].

### Animal models and pathophysiology

Limited access to muscle biopsies hinders extensive investigations of the specific cellular mechanisms leading to the development of the muscle pathology, and *in vitro/ex vivo *cellular systems only partially reproduce the complexity of the tissue. The development of the first animal model of ColVI deficiency in 1998, engineered by invalidating the *Col6a1 *gene in mice, has proven central to understanding the cellular pathways involved in these diseases. Homozygous animals were reported to develop a mild myopathic phenotype, and were initially described as a model of BM [97]. Interestingly, the diaphragm was the most affected muscle, with signs of necrosis evidenced by uptake of Evan's blue dye [97]. Subsequently, a latent mitochondrial dysfunction accompanied by ultrastructural alterations of mitochondria and the sarcoplasmic reticulum, resulting in spontaneous apoptosis, was found in about one-third of muscle fibers [98]. Reduced contractile strength of the diaphragm and other muscle groups was also reported in *Col6a1^-/- ^*mice in this initial study [98]. The maximal isometric tension generated by ColVI-deficient skinned fibers from gastrocnemius was found to be reduced in a recent report; however, using a protocol of eccentric contractions *in vivo*, no muscle force drop was found, indicating that the lack of ColVI does not impair myofibrillar function [99]. Importantly, mitochondrial dysfunction was also reported in cultured muscle cells from patients and could be reversed by cyclosporin (Cs)A, an immunosuppressive drug that prevents the opening of the mitochondrial permeability transition pore through binding to cyclophilin D, and also inhibits the phosphatase calcineurin [100,101]. Another *in vitro *study showed that patients-derived skin fibroblasts behave differently from myoblasts in that respect, and also questioned the specificity of this mitochondrial dysfunction [102], warranting further studies on the matter.

A role for cell survival had previously been proposed for ColVI because it was shown to prevent anti-α1 integrin-mediated apoptosis and trigger the downregulation of bax, a pro-apoptotic molecule [103,104].

**Figure 5:**
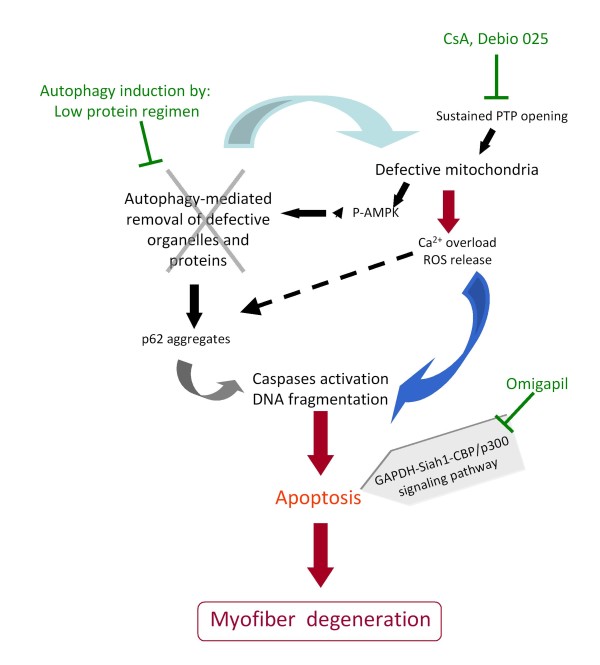
**Current pathological hypotheses and therapeutic targets**. The currently known cascade of main events leading to myofiber degeneration in ColVI-deficient skeletal muscle is shown. Mitochondrial dysfunction (due in part to the defective permeability transition pore (PTP) opening) triggers an energetic imbalance with the increased levels of phosphorylated adenosine monophosphate-activated protein kinase (p-AMPK), Ca^2+ ^overload and the production of reactive oxygen species (ROS). Lack of autophagy induction exacerbates the cellular dysfunction because defective mitochondria and proteins (such as p62 aggregates) are not cleared from the cytoplasm. Together, these defects lead to increased apoptosis. Potential therapeutic interventions are indicated in green.

Recently, a study of the autophagic process in muscles of *Col6a1 *knockout mice revealed that autophagy was not induced efficiently [105]. The ensuing defective autophagy provides the link between the previously described mitochondrial dysfunction and myofiber degeneration, as abnormal organelles and molecules cannot be efficiently cleared from the cell. This study further showed that the forced induction of autophagy, either by dietary restriction or by treatment with rapamycin or CsA, ameliorated the phenotype of the *Col6a1^-/- ^*mice (Figure 5). A similar alteration of autophagy was also detected in muscle biopsies derived from nine patients with UCMD or BM [105]. These data thus provide a basis for novel therapeutic targets to promote the elimination of defective organelles in ColVI-deficient skeletal muscle.

Morpholino-mediated knock-down of the *col6a1 *and *col6a3 *genes in zebrafish embryos showed that collagen VI deficiency significantly impairs muscle development and function [106]. Increased apoptosis, partially prevented by CsA treatment, was also described in the zebrafish morphants [106]. As in other instances, perturbation of muscle components leads to a more severe phenotype in zebrafish than in mouse models, which may in part be due to intrinsic differences in muscle development in these species, especially in terms of timing. Zebrafish models have emerged as major *in vivo *models of neuromuscular disorders, and seem to be particularly well suited for whole-organism screens for potential pharmacological treatments, as recently illustrated in zebrafish models of Duchenne muscular dystrophy [107].

### Therapeutic intervention

To date, no curative treatment exists for these disorders, and most patients rely on supportive treatment of symptoms, usually involving orthopedic (spinal deformations, contractures) and respiratory complications [108].

The unveiling of mitochondrial dysfunction led to an open pilot trial in five patients with UCMD or BM treated orally with CsA for 1 month [109]. This study reported normalization of the mitochondrial dysfunction and decrease of apoptosis of muscle cells following this short-term treatment [110,111]. Longer treatment (up to 2 years) had some beneficial effect on muscle function in these patients but did not prevent progression of the disease in the children [38]. Debio-025 (D-MeAla3EtVal4-cyclosporin; DebioPharm) prevents the inappropriate opening of the mitochondrial permeability transition pore (PTP) without interfering with calcineurin [112], and was shown to restore mitochondrial function in cultured muscle cells of patients [100] and in *Col6a1^-/- ^*mice [113]. Debio-025 is currently being tested in a phase II clinical trial in patients with chronic hepatitis C. Another anti-apoptotic pharmacological agent that is being investigated in the context of ColVI myopathies is Omigapil (N-(dibenz(b, f)oxepin-10-ylmethyl)-N-methyl-N-prop-2-ynylamine maleate; Santhera Pharmaceuticals), a chemical derivative of (-)-deprenyl, which was shown to reduce GAPDH-Siah1-mediated apoptosis in a mouse model of laminin α2 chain deficiency [114].

It should be noted that translating some of this research from animal models to patients represents a challenging task, particularly because, to date, these drugs have not been approved for use in children, the patient population with the most severe forms of ColVI myopathies. In addition, there is concern about these therapeutic approaches because of the pleiotropic, and potentially harmful, consequences of anti-apoptotic and/or pro-autophagy treatments. Furthermore, such approaches aiming at modulating downstream pathways would not address the primary defect in these disorders, that is, lack of ColVI in the connective tissue, and would thus need to be continually administered. For the sake of discussion, several alternative, and not necessarily exclusive, therapeutic avenues that would sustain re-expression of ColVI may be envisioned. These approaches may consist of gene-based therapies, such as vector delivery of ColVI-coding sequence, and antisense inhibition of mutant transcripts exerting dominant-negative effects [96]. Additionally, as nonsense mutations leading to PTCs are often associated with early-onset, severe phenotypes [42], pharmacological approaches aiming to 'force' translation of PTCs (a phenomenon known as 'translational readthrough' [115]) may prove beneficial for a subset of patients carrying these types of mutations. However, the complex assembly process and regulation of ColVI may prove challenging and may limit the realistic options to be investigated.

## Conclusions

The past decade of research on neuromuscular disorders has proven very exciting, and has seen ColVI myopathies emerge as an important set of disorders, rather under-recognized until recently. Many challenges remain despite the tremendous advances in the understanding of their genetic, biochemical and pathophysiological bases. It is hoped that the decade(s) to come will see the development of safe and efficient therapies for these disorders. Consequently, as for other rare diseases, the scientific community, and patient organizations, and patients and their families have become increasingly aware of the need for databases, both clinical and genetic, to facilitate recruitment of patients for upcoming clinical trials.

## List of abbreviations used

BM: Bethlem myopathy; ColVI: collagen type VI; *COL6A*: gene(s) encoding the alpha chain(s) of collagen VI; CsA: cyclosporin A; DAPI: 4',6-diamidino-2-phenylindole; EDMD: Emery-Dreifuss muscular dystrophy; EDS: Ehlers-Danlos syndrome; *LMNA*: gene encoding lamin A/C; MDC1A: congenital muscular dystrophy with laminin α2 chain deficiency; PTP: permeability transition pore; MRI: magnetic resonance imaging; PTC: premature termination codon; ROS: reactive oxygen species; *SEPN1*: gene encoding selenoprotein N; *TNXB*: gene encoding tenascin-X; UCMD: Ullrich congenital muscular dystrophy

## Competing interests

The authors declare that they have no competing interests.

## Authors' contributions

All authors contributed to the writing of this manuscript. All authors read and approved the final manuscript.

## Authors' information

Valérie Allamand holds a PhD in Human Genetics, and currently leads a group focusing on ColVI myopathies in the research unit directed by Thomas Voit. In 2009, she co-organized, with Drs Kate Bushby and Luciano Merlini, the 166th ENMC International Workshop on Collagen Type VI-Related Myopathies (22-24 May 2009, Naarden, The Netherlands). She is the genetic curator of the UMD-*COL6 *databases, developed in the context of the Treat-NMD European network of excellence.

Laura Briñas obtained her PhD in Molecular Biology. She joined the Institut de Myologie in September 2006 as a post-doctoral fellow, and has been involved in the molecular analysis of mutations in the genes encoding collagen VI and the dissection of their cellular consequences.

Susana Quijano-Roy is a child neurologist with previous medical training in La Paz Hospital (Madrid, Spain) and Boston Children's Hospital (USA). She has held a clinical practice since 2001 at Garches Neuromuscular Reference Center (GNMH), and leads the pediatric EMG laboratory at Necker Enfants Hospital, Paris. She obtained her PhD in 2004 with a study on congenital muscular dystrophies (CMDs). She is part or the international expert group that is currently defining diagnosis, standards of care and natural history of CMDs and establishing outcome measures for future therapeutic trials.

Tanya Stojkovic is a medical doctor, specialized in neurophysiology and neuromuscular disorders. She has worked in the neuromuscular clinical unit directed by Professor Eymard since 2006 (Pitié-Salpêtière, Institut de Myologie, Paris, France). She is involved, as a clinician, in the diagnosis of neuromuscular disorders. She has a special interest in ColVI-related myopathies.

Pascale Richard holds a PharmD, Graduation from Medical Biologist and a PhD in molecular genetics. She is head of the 'Functional Unit of Molecular Cardiogenetics and Myogenetics' at Pitié-Salpêtrière Hospital in Paris, where she developed a functional unit focused on the molecular diagnosis of cardiomyopathies and congenital and progressive myopathies in close collaboration with the research units. This unit constitutes the only laboratory in France where the genetic diagnosis of ColVI myopathies is proposed.

Gisèle Bonne holds a PhD in developmental physiology, followed by a post-doctoral training in human genetics. She currently leads the team 'Genetics and Pathophysiology of Neuromuscular Disorders' in the research unit directed by Thomas Voit at the Institut de Myologie in Paris. Her research group focuses on neuromuscular disorders caused by mutations in the lamin A/C gene.
